# Modulation of Metabolome and Overall Perception of Pea Protein-Based Gels Fermented with Various Synthetic Microbial Consortia

**DOI:** 10.3390/foods11081146

**Published:** 2022-04-15

**Authors:** Salma Ben-Harb, Anne Saint-Eve, Françoise Irlinger, Isabelle Souchon, Pascal Bonnarme

**Affiliations:** 1 Université Paris Saclay, INRAE, AgroParisTech, UMR SayFood, F-78850 Thiverval Grignon, France; salmabharb@gmail.com (S.B.-H.); anne.saint-eve@inrae.fr (A.S.-E.); francoise.irlinger@inrae.fr (F.I.); isabelle.souchon@inrae.fr (I.S.); 2 INRAE, Université d’Avignon UMR SQPOV, F-84000 Avignon, France

**Keywords:** beany notes, bitter, aroma compounds, fermentation, sensory analysis, olfactometry

## Abstract

Moving to a more sustainable food system requires increasing the proportion of plant protein in our diet. Fermentation of plant product could thus be used to develop innovative and tasty food products. We investigated the impact of fermentation by synthetic microbial consortia (SMC) on the perception of pea protein-based gels, giving possible keys to better understand the origin of sensory perception (e.g., beany, bitter). Two types of pea gels, containing (i) 100% pea proteins and (ii) 50% pea proteins/50% milk proteins, were fermented with three different SMC. Major species developing in both types of gels were *Geotrichum candidum*, *Lactococcus lactis*, and *Lactobacillus rhamnosus*. In pea gels, sensory analyses revealed that bitterness increased after fermentation, which could be due to hydrophobic amino acids resulting from protein hydrolysis, but also decreased pea note intensity in pea gels. In mixed gels, pea perception was similar whatever the SMC, whereas cheesy perception increased. Olfactometry experiments revealed that some specific “green” aroma compounds, responsible for green off-note, were suppressed/reduced by fermentation. The data presented investigated to which extent the design of SMC, together with gels composition (pea gels versus mixed gels), could modulate sensorial perception and drive consumer acceptability.

## 1. Introduction

Pea (*Pisum sativum* L.) is an important vegetable source of proteins and could become a potential alternative to soybean in Europe [1]. However, and similarly to other legumes, pea off-flavors can constitute a barrier for consumers and may limit the use of pea proteins into mainstream food applications [2]. Unpleasant taste and aroma perceptions were identified in numerous studies, with consequences on consumer acceptance. In particular, beany aroma and bitter taste are described as potential barriers in food application. They are partially constitutive to peas and may be modulated through harvesting, storage, and processing [3].

Beany off-flavor, already described in many legumes such as soybeans or peas, is defined as a complex olfactory perception [3,4,5,6,7]. It could include the perception of musty/earthy, musty/dusty, sour aromatics, starchy, powdery feel, and also green/pea pod, nutty, or brown [3,4,7]. In the literature, the main components identified as responsible for these undesirable beany odors belong to various families, such as aldehydes, ketones, and alcohols [7]. Sulphur compounds and aromatic hydrocarbons could also contribute to beany perception [2]. As examples, the most common cited compounds associated with beany perception are: 3-methyl-1-butanol; pentanol; 1-octen-3-ol; trans, trans-2,4-heptadienal; acetophenone; 1-octen-3-one; 3-isopropyl-2-methoxypyrazine. Other chemicals, such as hexanal, trans-2-hexenal, trans-2-octenal, and pentanal, commonly found as volatiles of soy products, are also described as beany, depending on their concentration [7].

Bitterness is widely described as a sensory off-note in many plant products. It is often attributed to the release of low molecular weight peptides containing hydrophobic amino acids residues, particularly leucine, proline, phenylalanine, and tyrosine [8,9]. The hydrophobicity, primary sequence, spatial structure, molecular weight, and bulkiness of peptide has been also studied as possibly influencing bitterness [10]. Pea protein bitterness could also be related to the saponin content, mainly dependent on the pea variety [11], and on the method used for proteins extraction [12]. Besides isoflavones and flavonoids [13], isochlorogenic and chlorogenic acid [14] have also been identified as responsible for bitterness. Finally, compounds coming from lipid metabolism and oxidation have also been identified as responsible for bitter perception in pea protein isolates [15]. To increase the potential use of pea proteins in food, it seems necessary to develop some applications in which the process can reduce, mask, or eliminate off-notes, relative to bitterness and beany flavor. Fermentation could be a solution, as already highlighted in pea products fermented by lactic acid bacteria (*Lactobacillus plantarum, Pediococcus pentosaceus, Streptococcus thermophilus, Lactobacillus acidophilus, Streptococcus thermophilus, Lactobacillus helveticus, Lactobacillus delbrueckii, Lactobacillus rhamnosus, Lactobacillus fermentum*) [6,16], a combination of lactic acid bacteria (VEGE 047 LYO, DuPont Danisco) and yeasts (*Kluyveromyces lactis, Kluyveromyces marxianus, Torulaspora delbrueckii*) [17] or SMC (*Lactobacillus rhamnosus, Leuconostoc lactis, Geotrichum candidum, Candida catenulate, Yarrowia lipolytica, Brevibacterium antiquum, Brevibacterium casei, Hafnia alvei*) [18,19].

Microorganisms such as *Lactobacillus* strains are extensively used as debittering starter adjuncts for the production of protein hydrolysates deprived of bitter taste [20]. Similar effects have been observed during hydrolysis with fungal (e.g., *Rhizopus oryzae, Aspergillus oryzae, Actinomucor elegans, Aspergillus sojae*) proteases preparations and microbial aminopeptidases hydrolyzing bitter peptides and liberating aromatic amino acids (also responsible for bitterness), which are also important precursors of aroma compounds [9].

To date, only a few investigations have been performed on the effect of fermentation, by using SMC, on the sensory characteristics of pea protein-based products with a high pea content. Thus, this study focused on the impact of the fermentation process on the perception of different fermented pea-based gels. We chose to focus on the relative effect of different SMC so that to evaluate their ability to modify the perception of pea gels, especially in the modulation of beany flavor and bitter taste. To go further in our understanding, the molecules possibly responsible for the sensory perception were determined by the identification of the composition of volatile and non-volatile compounds.

## 2. Materials and Methods

### 2.1. Raw Materials

Pea protein isolate (PPI) (NUTRALYS^®^ S85F) was provided from Roquette Frères (Lestrem, France). Skim milk powder (SMP) was purchased from Lactalis (Bourgbarré, France) and Rapeseed oil (Fleur de Colza, Lesieur, France) was purchased from a local supermarket. Glucono delta-lactone (GDL, Sigma Aldrich, Steinheim, Germany) was used for coagulation. Fifteen strains from different taxonomic order of bacteria (*Actinobacteria, Firmicutes, Proteobacteria*) and yeasts (*Saccharomycetales*) used in this study were isolated from dairy and vegetable products and were obtained from the private collection of the Sayfood Unit or from different international collections (ATCC, the American Type Culture Collection (Manassas, VA, USA); CIP, Collection of Institut Pasteur (Paris, France); BCCM/LMG, Laboratory of Microbiology, Department of Biochemistry and Microbiology (Ghent, Belgium); NCDO, National Collection of Dairy Organisms, Aberdeen, United Kingdom; NCYC, National Collection of Yeast Cultures (Norwich, UK); CLIB, Collection de Levures d’Intérêt Biotechnologique (Montpellier, France) (Table S1).

Two types of microbial consortia were designed from previous works [18,19]. The first one is VEGAN consortium: it was selected for its good performance on pea protein matrices. The second one is MEGAN consortium: it was selected for its good performance on mixed pea/milk protein matrices. To investigate a possible effect of strains origin on their adaptation potential to the matrix (pea or mixed gels), two MEGAN consortia were designed with the same microbial species from different origin: MEGAN-A (A = animal) was composed of strains isolated from dairy products, while MEGAN-V (V = vegetable) was composed of strains isolated from plant.

### 2.2. Products Preparation, Microbial Analyses, and pH Measurements

Two types of fermented gels were prepared in the same conditions, containing a total of 10% of proteins, with (i) 100% pea proteins (Pea gel), or (ii) a mixture of pea proteins and milk powder (50/50) (Mixed gel). Four fermented gels (Pea gels fermented with VEGAN and MEGAN-V consortium, and mixed gels fermented with MEGAN-A and MEGAN-V consortium) were prepared according to their best growth performance on one or two matrices and fermented as previously described by [19]. Control (non-fermented) gels were prepared in the same conditions. All gels were prepared in triplicate.

The gels were formed by chemical coagulation using GDL (glucono delta-lactone). To obtain gels, suspensions were mixed with GDL (0.1 and 0.5 *w*/*v* for pea and mixed emulsions, respectively) and with the different microbial consortia with a cell density equivalent to 6.0 log CFU/g for each bacterial strain and 4.0 log CFU/g for each yeast. The mixtures obtained were then incubated at 25 °C for 24 h for coagulation and at 16 °C for 2 days for fermentation [19].

After fermentation, fermented gels were mixed using a strict protocol. Serial dilutions were prepared in 0.9% NaCl from one gram of gel and plated in triplicate on agar, as previously described by [18,19]. Fermentations were carried out in triplicate from independent batches, and data for pH and growth values were averaged. Distribution and growth were expressed as log N/N0 (N: growth of strains at three days of fermentation; N0: initial cell density). The pH values were the arithmetic means of three measurements made with a Blue Line 27 surface electrode (Schott).

The fermented gels were stored at −20 °C during the study, before analyses of volatile and non-volatile compounds by GC-MS and olfactometry and sensory analyses. For sensory analyses, all samples were then evaluated at 20 °C.

### 2.3. Sensory Evaluation

The sensory evaluation was performed by a sensory profile to evaluate the perception of fermented and non-fermented gels (control sample) by a trained panel. 

#### 2.3.1. Sensory Evaluation Conditions

A panel of ten volunteers (7 women and 3 men) were recruited, based on their motivations for participating. All panelists gave their free and informed consent and received compensation for their participation. Panelists were not trained on this product category prior sensorial evaluation, but were trained for this specific sensory study on the evaluation of plant-based products. They were asked not to eat or drink for at least 1 h before the study sessions. 

All sensory analyses were carried out in an air-conditioned room (20 °C), in individual booths, under white light. Samples were labeled with randomly selected three-digit numbers and 20 mL of sample were served at 10 °C in a 50 mL hermetically-sealed cup. The samples were balanced following a Williams’ Latin square experimental design order across panelists, taking care to avoid carry-over effects. Panelists were provided with mineral water (Evian, Danone, Evian les Bains, France) and a piece of apple to rinse their mouth and to limit persistence between samples. All samples were analyzed in triplicate.

#### 2.3.2. Attribute Selection, Panelist Training, and Sensory Profiling Evaluation

First of all, generation and selection of attributes was performed by discussion with the panel and conducted to the final reduced list of six attributes: bitter, pea, roasted/grilled, cheese/rind, fresh cream/butter, and fruity. Training sessions were proposed to panelists, and focused on attributes definition, ranking, and intensity evaluation. For session evaluation, each of these six attributes were evaluated for the six samples (two control gels: pea control and mixed control and four fermented gels: VEGAN-Pea gel, MEGAN-V pea gel, MEGAN-A mixed gel, MEGAN-V mixed gel) by using a 10-point unstructured intensity scale and Fizz Acquisition software (Version 2.47A, Biosystemes, Couternon, France). Samples were presented in monadic sequential mode in duplicate. A set of six products by session was proposed, loading to a total of four sessions of evaluation. At the end of the profiling assessment, the panel performances were validated in terms of global and individual homogeneity, discrimination ability, and repeatability, using XLStat software (Version 2010.4.02, Addinsoft, Paris, France).

### 2.4. Physicochemical Characterization

Volatile and non-volatile compounds in samples were identified by Gas-Chromatography coupled to Olfactometry and by ultra-high performance liquid chromatography.

#### 2.4.1. Extraction and Identification of Volatile Flavor Components from Fermented Gels

GC-Olfactometry experiments were conducted on four types of fermented gels, VEGAN pea gel, MEGAN-V pea gel, MEGAN-A mixed gel, and MEGAN-V mixed gel, and on two non-fermented control gels: pea control and mixed control. 

In order to study the impact of SMC on the composition of volatile flavor compounds, gas chromatography was performed on fermented gels. Each gel—pea gel or mixed gel—was fermented by two SMC (=the most adapted to the gel). For pea gels, control samples VEGAN gel and MEGAN-V gel were analyzed, and for mixed gels, control samples MEGAN-A gel and MEGAN-V gel were analyzed, conducting to six different products. All samples were analyzed in triplicate.

For all tests, samples were diluted 1/10 with Milli-Q water (Merck Millipore, Merck KGaA, Darmstadt, Germany) at 4 °C. Five milliliters of the mixture were homogenized using a Polytron© PT 2100 (VWR, Radnor, PA, USA; 10,000 g × 3 S), and were placed in a water-jacketed purge and trap apparatus (purge and trap concentrator, Tekmar-Dohrman 3100, Tekmar, Mundelein, IL, USA) at 40 °C (purge: 40 °C/15 min; Desorb: 225 °C/2 min) coupled to a gas chromatograph (Agilent Technologies 3800, Santa Clara, CA, USA) and a mass spectrometer detector (MSD 5975C, Agilent Technologies, Santa Clara, CA, USA). The apparatus was equipped with a polar capillary column (DB-WAX5 Polyethylene glycol 30 m × 0.25 mm; film thickness of 0.25 µm; Agilent 122-7032, Agilent, Santa Clara, CA, USA). Oven temperature increased from 40 °C to 60 °C at a rate of 1 °C/min then to 110 °C at a rate of 2 °C/min and to 220 °C at a rate of 20 °C/min.

Eight to ten panelists were selected from the sensory panel to perform olfactometry measures based on their motivation and availability. These panelists were already familiar with olfactometry analyses. They were instructed to signal each odor perceived throughout the sniffing session by pressing a push button. They were asked to verbally describe the perceived odor. Sniffing sessions lasted 25 min. 

The percentage of detection frequency was calculated for each compound (number of persons who detected the odorant compound/total number of panelists) × 100. All analyses (for each product and each panelist) were conducted in duplicate. 

Thus, two aromagrams and two chromatograms were obtained per panelist and per sample. Recording of vocabulary items was launched at the “start of injection” of the sample into the chromatography column. Olfactory classes were defined during data processing in order to highlight the key-odor zones of an aromagram. The identification of the volatile aroma compounds, corresponding to the odor perceived by the panelists, was conducted by comparison of GC–MS and GC–olfactometry experimental retention indexes, then by comparison between theoretical and experimental description. 

#### 2.4.2. Extraction and Identification of Non-Volatile Components from Gels

To identify free amino-acids in gels, ultra-high performance liquid chromatography (LC-MS) was performed. Samples were diluted 1/5 with cold Milli-Q water (MerckMillipore, Merck KGaA, Darmstadt, Germany). The mixture was homogenized using a Polytron© PT 2100 (VWR, Radnor, PA, USA) and was centrifuged for 10 mn at 10,000× *g* at 4 °C. Supernatant was poured on Vivaspin pipe (filtration at 10,000 Dalton) and centrifuged for 30 mn at 8000× *g* at 4 °C. Filtrate solution was diluted at 1/10 with ultra-pure water quality HPLC-MS with 1% of LC MS formic acid.

These analyses were performed with an ultra-high performance liquid chromatography (Ultimate 3000, Thermo Fisher, Waltham, MA, USA) coupled with a high-resolution mass spectrometer (hybrid quadruple—Orbitrap, Q-Exactive) on a Hypersil Gold Phenyl column (25903-152130 Thermo Fisher, 150 mm × 2.1 mm; granulometry 3 µm, oven temperature 25 °C). The mobile phase used was composed of ultra-pure quality water HPLC-MS, of perfluoropentanoic acid (3 mM) and of acetonitrile quality HPLC-MS (0.25 mL/min, 120 bar, 14 min). Detection was realized with a diode array detector (scanning from 200 to 400 nm), a complete spectrum in positive ionization and a range of masses between 60/850 u.m.a. The HPLC-MS data were analyzed with Trace Finder (Thermo Fisher) software using retention time and the ratio m/z.

### 2.5. Statistical Analysis

Data analysis was performed using XLSTAT (Addinsoft, Paris, France, 2017) and Fizz Data Treatment (Biosystemes^®^ 1999). For each sensory attribute, an ANOVA with product and subject as main effects and their interaction was performed to investigate the performance of the panel and to determine which attributes can significantly discriminate the products. When significant differences between products were revealed (*p* < 0.05), mean intensities were compared using the Newman–Keuls multiple comparison test to form different groups of products. Principal Component Analysis (PCA) was also performed on average data. In addition to this, a Factorial Multiple Analysis (FMA) was performed to analyze the sensory profile and the initial volatile fingerprints as subsets of variables. The RV coefficient was calculated between the first two axes of the samples’ partial configuration to analyze similarities between samples.

## 3. Results and Discussion

### 3.1. Microbial Growth after Fermentation

**Figure 1:**
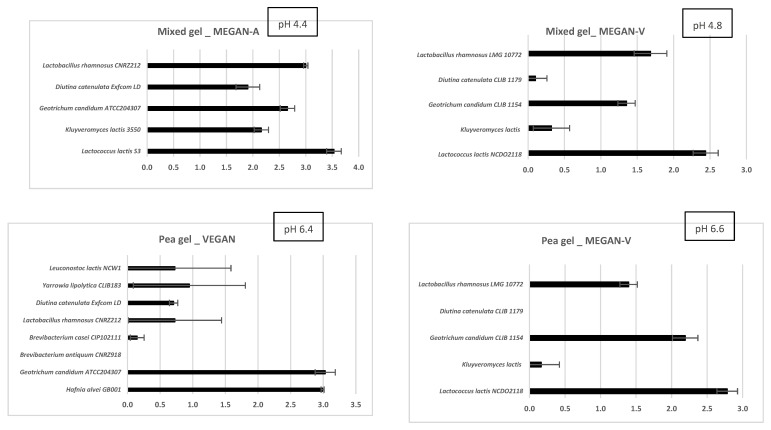
Growth (expressed as logN/N0, N: growth of strains at three days of fermentation; N0: initial cell density) of microbial species and pH values during the fermentation of pea gel and mixed gel (pea and milk) by three microbial communities (VEGAN, MEGAN-A, and MEGAN-V).

The populations reached in both types of gels after three days of fermentation are shown in Figure 1. The pH of pea gels fermented with VEGAN and MEGAN-V consortia remained fairly stable with respective values of 6.4 and 6.6, whereas the transformation of lactose into lactic acid by LAB led to significant acidification (pH 4.4 and 4.8) in the mixed pea/milk gel inoculated by MEGAN-A and MEGAN-V consortia. 

#### 3.1.1. Pea Gel

The pea gel inoculated with the MEGAN-V consortium, whose strains are of plant origin, is dominated by *Lactobacillus rhamnosus, Lactococcus lactis*, and *Geotrichum candidum.* The other two yeast strains—*Kluyveromyces lactis* and *Diutina catelulata*—did not colonize pea gel. The pea gel inoculated with the VEGAN consortium composed of strains isolated from dairy products, and previously selected for their aromatic capabilities on this gel [18,19], is dominated by two species of the MEGAN-V consortium (*Lactobacillus rhamnosus* and *Geotrichum candidum*) but also by other species (*Hafnia alvei, Leuconostoc lactis, Diutina catelulata, Yarrowia lipolytica*).

#### 3.1.2. Mixed Gel

The mixed gel inoculated with MEGAN-A, composed of strains of dairy origin, is dominated by all the strains of the consortium, *Lactococcus lactis, Lactobacillus rhamnosus, Geotrichum candidum, Kluyveromyces lactis*, and *Diutina catenulata*. The mixed gel inoculated with the MEGAN-V consortium is dominated by three species of the MEGAN-A consortium (*Lactococcus lactis, Lactobacillus rhamnosus*, and *Geotrichum candidum)* with the exception of *Diutina catelulata* and *Kluyveromyces lactis*. Whatever the consortium, the fermentation causes a strong acidification of the medium due to the production of lactic acid via the fermentation of lactose by lactic acid bacteria.

### 3.2. Flavor Description of Fermented and Non-Fermented Gels

**Figure 2:**
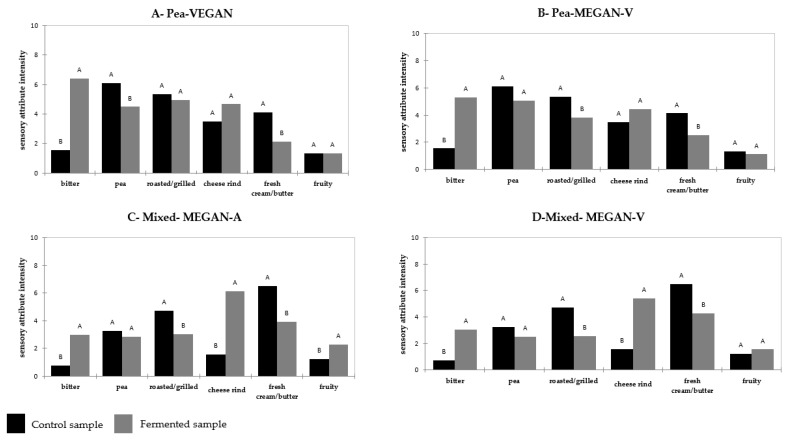
Average intensities of the sensory attributes evaluated during descriptive sensory analysis by panelists for pea and mixed gels. Fermentation effect between control sample (not fermented pea gel) and fermented sample (after three days of fermentation) were determined by ANOVA on each sensory attribute. Letters A and B indicate means that significantly differ between products at *p* < 0.05 (Newman–Keuls test). (**A**) Pea gels fermented by VEGAN consortium, (**B**) Pea gels fermented by MEGAN-V consortium, (**C**) Mixed gels fermented by MEGAN-A consortium, (**D**) Mixed gels fermented by MEGAN-V consortium.

The impact of fermentation on sensory characteristics was analyzed. Sensory profiles showed that fermented and non-fermented pea gels were perceived significantly differently for bitter, pea, and fresh cream attributes (Figure 2). ANOVA results showed that all fermented pea gels were characterized by a higher intensity in bitterness and a lower intensity of fresh cream perceptions than those of the pea control (*p* < 0.05). Pea perceived intensity significantly decreased after three days of fermentation for VEGAN consortium. Fruity and cheese-rind perceptions were not modified by fermentation, whatever the consortia used. Compared to the control sample, roasted/grilled notes were only perceived as lower in the MEGAN-V fermented gels.

For mixed gels, the intensity of the perception between fermented and non-fermented gels was different in bitterness, cheese rind, and fresh cream notes (Figure 2). Fermented mixed gels were perceived to be more intense as bitter and cheese rind, and less intense in fresh cream than the non-fermented mixed control. Moreover, in fermented mixed gels, the pea notes were perceived similarly to the control sample. In contrast, the fruity notes increased in MEGAN-A fermented gels compared to the control sample and remained unchanged in MEGAN-V fermented gels.

In the literature, fermentation was associated with a higher intensity in bitterness [21]. This perception could be due to the inherent presence of sapid glycosylated compounds such as saponins, isoflavones, flavonols, and phenolic acids [22].

The perception of the above-mentioned attributes could have been modified and can be modulated by the presence of molecules generated during fermentation. To obtain a better understanding of the sensory modifications, the volatile and non-volatile profiles were analyzed.

### 3.3. Description of the Volatile Compounds Identified from GC–O Analyses

To obtain a better insight into the impact of fermentation on the volatile profile, GC–O analyses were performed on the uninoculated and fermented samples. The objective of these measurements was to highlight key aroma compounds generated in fermented gels for each type of matrix, compared to non-fermented gels. The results showed no repetition effect—with a threshold of 5%—for all molecules, whereas significant differences between the fermented samples in comparison to non-fermented gels were observed (*p* < 0.05) (Table S2). We chose to present only the main odorant events, which presented more than 65% of the percentage of detection.

In pea control sample, seven main odorant zones (out of twenty-four odorant events in total) were highlighted and could be linked to volatile molecules (Table 1). All odorant zones were mainly illustrated by vegetable and roasted/grilled notes. The most perceived zones, in terms of citation frequency, were linked to hexanal, detected by 100% of the panelists and described as vegetal notes; 3-methylbutanal, detected by 85% of the panelists, was mainly described as roasted/grilled notes; propanal was detected by 70% of panelists and described by vegetal and/or lactic notes; pentanal was also detected by 70% of panelists and described as roasted/grilled notes. Acetaldehyde, butanal, and propan-2-one were detected by 65% of panelists and described as lactic, vegetal, and roasted/grilled notes, respectively. In pea-VEGAN gels, four main odorant zones (seventeen odorant events in total) could be linked to volatile molecules. In terms of frequency, the most perceived zone is associated with lactic notes due to the presence of acetaldehyde detected by 100% of the panelists, and 3-methylbutanal was perceived by 85% of panelists and described as roasted/grilled. Roasted/grilled notes were also perceived (85% of the panelists) by the presence of 2-methylbutanal and propan-2-one. In pea-MEGAN-V gels, four main odorants zones (eleven odorant events in total) could be linked to identified volatile molecules. These compounds were globally described by different odors such as fruity, lactic, and roasted/grilled notes. The most perceived zones in terms of frequency were linked to 2-methylpropanal and ethyl-2-methylpropanoate detected by all panelists and described as roasted/grilled and fruity notes, respectively; pentan-2-one was detected by 94% of panelists as lactic notes and ethyl 3-methylbutanoate was detected by 88% of panelists as fruity notes.

Compared to the pea control, the perception of green notes in VEGAN and MEGAN-V fermented pea gels decreased considerably due to the disappearance of molecules responsible for beany notes such as hexanal and butanal. Interesting notes appeared, such as fruity and lactic notes. Some molecules were perceived differently between both samples, most probably due to a variation of their concentration in gels; for example, 3-methylbutanal was perceived as roasted/grilled and vegetal in pea control and only roasted/grilled in VEGAN-gel. These differences could be due to an increase in the concentration of 3-methylbutanal during fermentation together with the presence of other compounds produced during the fermentation process, and also a change in the composition/texture of the matrix resulting from fermentation.

Table 2 shows results associated with mixed gels. In the mixed control gel, four odorant zones (seventeen odorant zones in total) could be linked to volatile molecules. These compounds were described by different types of odor mainly characterized by roasted/grilled and vegetal notes. The most perceived event, in terms of frequency of citation, was linked to hexanal detected by 95% of the panelists and described by vegetal notes, and 2-methylbutanal and 3-methylbutanal were detected by 85% of panelists mainly as vegetal notes, and as lactic notes to a lower extent. Other notes also appeared as roasted/grilled and fruity due, for instance, to the presence of propan-2-one (84% of panelists). In the MEGAN-A mixed gel, five odorant zones (thirteen odorant zones in total) could be linked to volatile molecules. The most perceived event was linked to butane-2,3-dione detected by all panelists and described by butter notes. Both molecules, 2-methyl butanal and 3-methylbutanal, were perceived as lactic notes in these fermented gels, although they were perceived as vegetal notes prior to fermentation. In the MEGAN-V mixed gel, five odorant zones (ten odorant zones in total) could be identified with volatile molecules. The most perceived zones, in terms of frequency of citation, were linked to 2-methyl propanal, detected by 100% of panelists and described as roasted/grilled notes; 2-methylbutanal or 3-methyl butanal detected by 93% of panelists and also described as roasted/grilled notes. Fruity notes were perceived and could be due to the presence of ethyl-2-methylpropanoate and methyl disulfanyl methane. Butane-2,3-dione was also detected by 93% of panelists and described as lactic notes.

After fermentation, the perception of green notes decreased considerably, and interesting notes associated to fruity and lactic descriptors appeared. Previous studies showed that lactic fermentation could eliminate compounds associated with the beany flavor of soymilk, such as hexanal and 2-pentylfuran [23,24]. Moreover, lactic fermentation with *Lactobacillus plantarum* or with *Pediococcus pentosaceus* could decrease the concentration of hexanal in lupin protein extracts [25].

The control gels were mainly characterized by the presence of aldehydes, affecting the overall perception to give “green notes”. Aldehydes could be derived from either enzymatic or auto-oxidation of fatty acids—mainly linoleic and linolenic acids—present in peas [3]. Hexanal was characterized by a fatty, green, grassy odor in pea and mixed gels [5]. Thus, the perception of the green notes could arise from the interactions between different molecules and would, therefore, not be the result of the perception of one single compound [26].

The fermentation of gels led to a significant reduction in the total area counts of aldehydes with green sensory descriptors (Table S2). Besides, some volatile compounds were generated following fermentation. This is typically the case of 2-methylbutanal in VEGAN-pea gels—arising from isoleucine catabolism [27]—which has malt, chocolate, and roasted coffee notes. The presence of roasted/grilled notes in the fermented pea gels could also be associated with the proteolysis of vicilin in pea proteins by the action of microorganisms. It has been shown that peptides resulting from proteolysis of vicilin-like globulin of cocoa seeds could be responsible for the formation of the cocoa-specific aroma components during the roasting process. Such components are generated from the hydrolysis of cocoa vicilin by the cooperative action of an aspartic endoprotease and a carboxy peptidase present in ungerminated cocoa seeds [28]. Considering MEGAN-V consortium, fermentation of pea or mixed gels revealed the accumulation of esters (Table S2), such as ethyl-2-methylpropanoate, ethyl 3-methylbutanoate due to the growth of microorganisms such as yeasts. Esters are well known to contribute to typical fruity notes in fermented products [29], and 3-methylbutanoate is an acid commonly found in peas, soybean, and other common food products (e.g., milk, fruits, cheese). This acid can be produced as a microbial metabolite during the leucine metabolism [30]. The presence of ethyl 3-methylbutanoate was previously reported [31] during the fermentation of lupin wheat flour. In agreement with this, preliminary studies [32] showed that 3-methylbutanoic acid, naturally present, considerably increased upon storage of the lupin seeds, probably due to microbial spoilage through leucine catabolism.

Olfactory perception is largely impacted by the quality of proteins, in particular by the interactions between pea proteins and volatiles. These interactions can be related to the solubility of proteins [33], and the composition in amino acids and peptides, which can have consequences on perceived aroma intensities [34]. 

### 3.4. Identification of Non-Volatile Components from Fermented Gels

**Figure 3:**
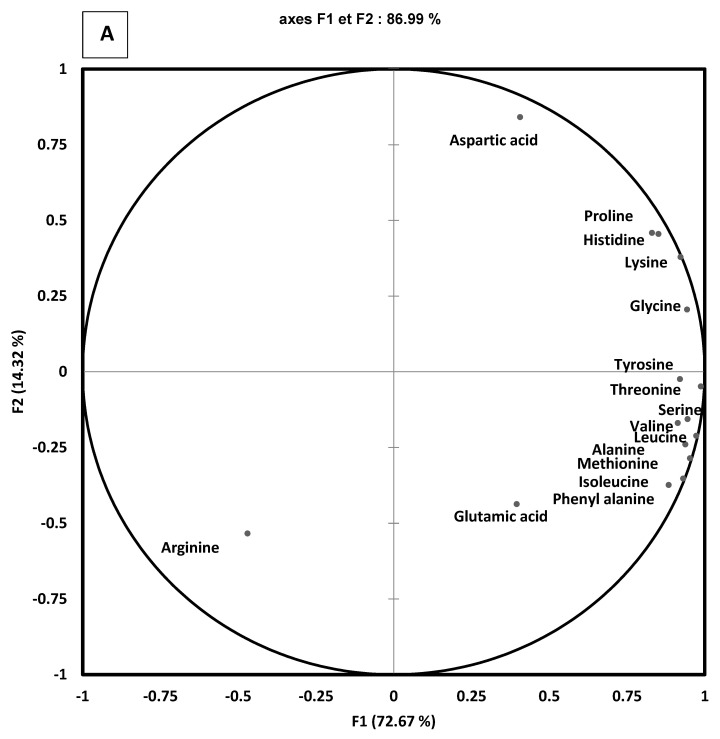

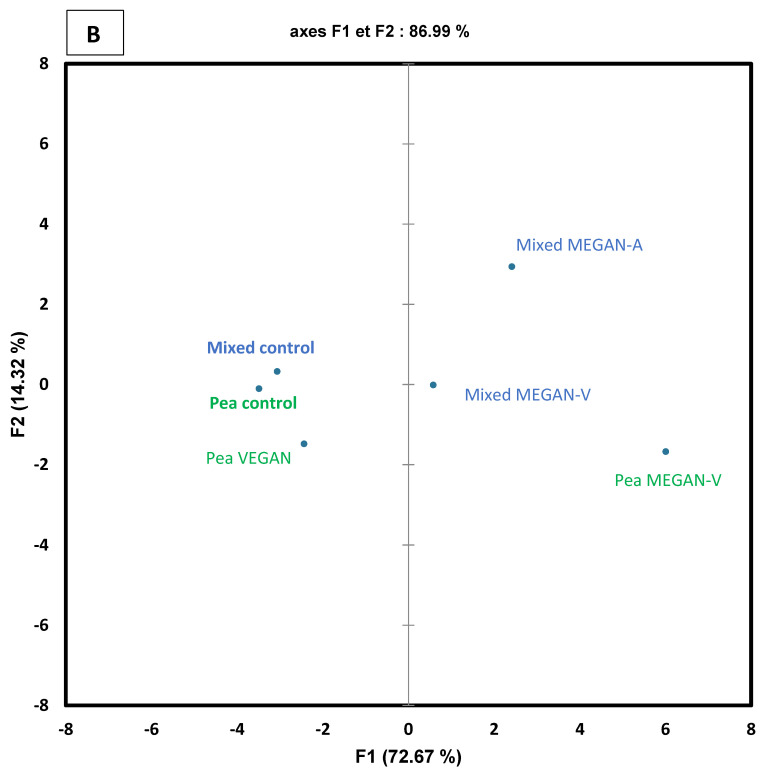
Free amino acid composition of pea and mixed gels: not fermented gel and fermented gel at three days with the different microbial consortia: VEGAN (VEG), MEGAN-A (MEG-A), and MEGAN-V (MEG-V). (**A**) map of amino acids composition, (**B**) map of products: Blue: Mixed gels, Green: Pea Gels.

Sixteen free amino acids were identified by LC-MS in fermented and non-fermented gels. To illustrate their occurrence in the different fermented gels, a Principal Correspondence Analysis (PCA) was performed (Figure 3). The first axis of the map, explaining 72.67% of the total variance, could separate pea control, mixed control, and pea-VEGAN from the other fermented gels (Mixed-MEGAN-A, Mixed-MEGAN-V, and Pea-MEGAN). 

Except for the pea-VEG gels and control pea gels, characterized by a low number of amino acids and loaded negatively on the F1 and/or F2 axis, the Pea-MEGAN-V gels were characterized by high quantities of amino acids such as phenylalanine, leucine, valine, serine, alanine, and isoleucine. 

The fermented mixed gels could be clustered in two groups. The first group included mixed-MEGAN-V and was characterized by a low concentration of amino acids similar to the control sample. The second group of products, composed of Mixed-MEGAN-A, was characterized by a high amount of free amino acids like proline, lysine, and histidine. These results suggest that the origin of the strains greatly impact the fermentation process and the amino acids composition following protein degradation. This composition greatly depends on the proteolytic potential of microorganisms, which hydrolyze proteins into peptides and amino acids [35].

### 3.5. Relationships between Sensory Characteristics, Volatile, and Non-Volatile Components in Fermented Gels

**Figure 4:**
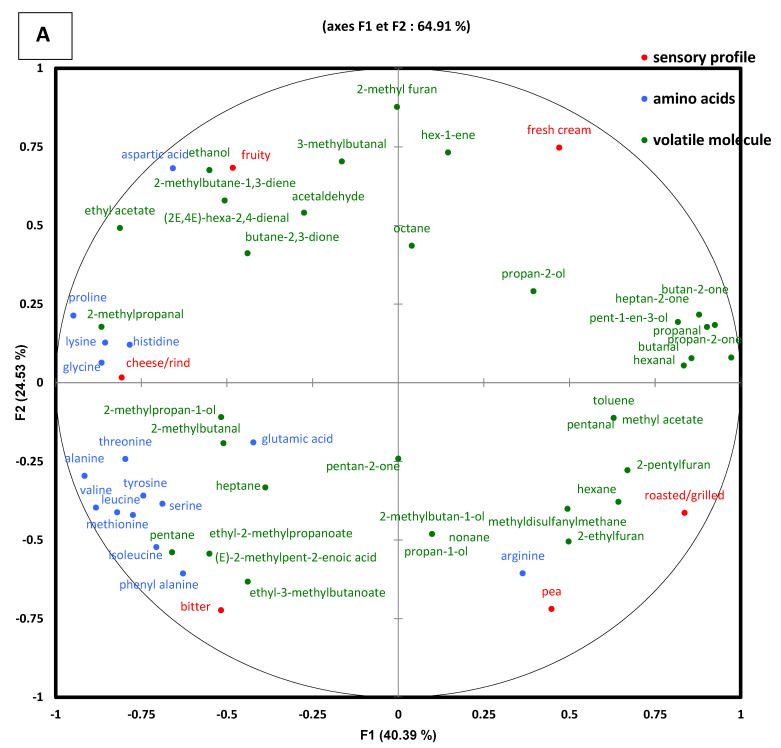

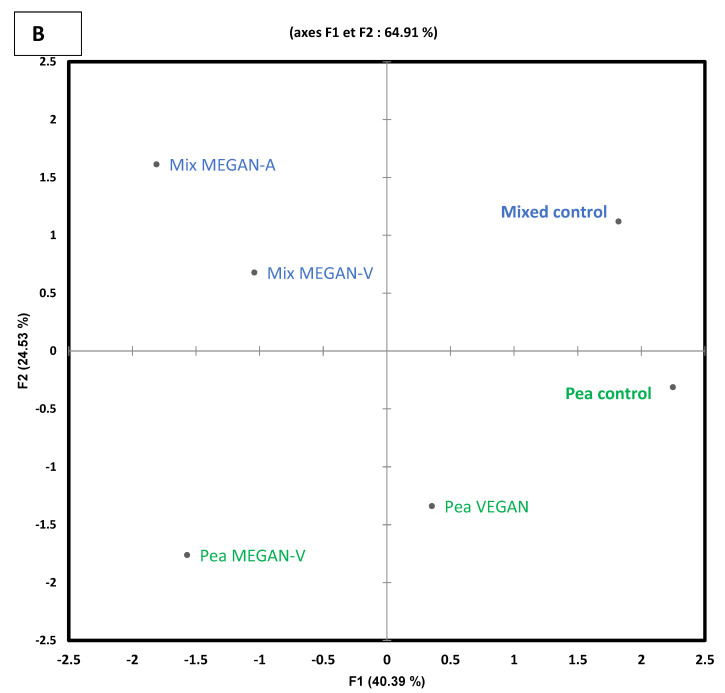
MFA analyses performed with sensory variables, volatile compounds, and amino acids composition of the different pea and mixed gels. Red: sensory attributes, Green; volatile compounds, Blue; amino acids. RV coefficients were (sensory attribute-volatile compounds = 0.77)/(sensory attribute–amino acids) = 0.45)/(amino acids—volatile compounds = 0.72). (**A**) map of sensory attributes, volatile compounds, and amino acids composition, (**B**) map of pea (Green) and mixed (Blue) products (not fermented gel and fermented gel) at three days with the different microbial consortia: VEGAN, MEGAN-A, and MEGAN-V.

In order to study the relationships between sensory perceptions, volatile, and non-volatile components of pea and mixed gels, a Multiple Factorial Analysis (MFA) was performed (Figure 4). The first axis of the map, explaining 40.4% of the total variance, could separate pea control, mixed control, pea-VEGAN, and the other fermented gels (Mixed-MEGAN-A, Mixed-MEGAN-V, and Pea-MEGAN-V) (Figure 4B).

The first and second dimensions of this MFA of gels explained 64.9% of the information. The roasted/grilled notes, which loaded positively on the F1 axis and negatively on the F2 axis, were mainly associated with some volatile molecules like furan2-pentyl and furan2-ethyl. The fruity notes were loaded positively on the F2 axis and negatively on the F1 axis; they were associated with the presence of aldehydes (acetaldehyde; 3-methylbutanal; (2E,4E)-hexa-2,4-dienal), ethyl acetate, butane-2,3-dione, ethanol. Results show that cheesy rind notes were related to protein degradation as indicated by the presence of a variety of free amino acids. This most probably results from the proteolysis of the milk protein fraction of the mixed gels by MEGAN-A and MEGAN-V consortia. 

Our results corroborate those of [36]. These authors observed that the fermentation of lupin with lactic acid bacteria (*Lactobacillus delbrueckii* subsp. *bulgaricus* and *Streptococcus thermophillus*) increased mushroom, soil, green, and nutty aroma characteristics without decreasing beany odor. However, they observed a significant increase in the concentration of 1-penten-3-one, 1-octen-3-one, and 3-octen-2-one contributing to mushroom and soil odors. Their work highlighted that concentration of hexanal and (E)-2-octenal significantly increased after fermentation, leading to an increase in the perception of beany and green odor notes. However, the changes in other volatiles associated with mushroom, soil, and baked attributes may contribute to increase the overall acceptability as masking agents. 

Bitterness perception was mainly related to the presence of high quantity of hydrophobic amino acids like phenylalanine, leucine, valine, and isoleucine, which are generated through proteolysis during fermentation. This result is in agreement with the literature, since bitterness is often attributed to the release of low molecular weight peptides containing hydrophobic amino acid residues, particularly leucine and phenylalanine [8]. The hydrophobicity, primary sequence, spatial structure, molecular weight, and bulkiness of peptides have been studied as possibly influencing bitterness perception [10]. This may explain that the perception of bitterness in other samples such as mixed gels could be related to other factors. Pea fermented gels were perceived to be more intense in bitterness than mixed gels, probably due to the presence of a higher concentration of pea proteins in pea gels, together with the presence of milk protein which could modulate bitterness perception. Some replicates were performed and evaluated to confirm the stability of the sensory parameter with the fermentation conditions.

## 4. Conclusions

This study was conducted to investigate the effects of fermentation with different SMC and the impact of their origin on the sensory properties of pea-based gels. A number of changes occurred during fermentation, important ones being a modification in the composition of volatile and non-volatile compounds, with consequences on the sensory properties of the fermented gels. The origin of the strains (dairy versus animal origin) may also have an effect on these modifications. Aroma profile assessment revealed that roasted/grilled notes were characteristics of the pea-VEGAN gels, while pea-MEGAN-V gels were characterized by fruity and roasted/grilled notes, mixed-MEGAN-A gels by lactic notes, and mixed-MEGAN-V gels by fruity and roasted/grilled notes. A major result is that specific green notes of pea, associated with the presence of aldehydes such as hexanal, were significantly reduced by fermentation with SMC. In mixed gels, the cheesy notes increased, but the pea notes did not change after fermentation with all SMC. In pea gels, the pea notes decreased with VEGAN consortium. Besides, the bitterness perception increased during fermentation, whatever the SMC in all tested gels, but to a lesser extent in mixed gels. Mixed gels fermentation could therefore be an interesting option to reduce sensorial defects of pea, while keeping some familiar sensorial attributes such as those of dairy products. 

Creating new foods that combine animal- and plant-based ingredients should be promoted as part of the effort to design more sustainable and desirable foods. Some consumers are clearly interested in consuming plant-based or mixed products, as highlighted in previous work [37]. Fermentation is one of the different strategies which can be used to reduce the off-notes described in many protein-based products made from legumes such as peas, lupins, lentils, chickpeas, and common beans [2]. Besides, it may offer a wide spectrum of volatile profiles [6,9,17,19,25] and a potential source of new product innovation [37]. Furthermore, while most of the fermentation studies used pure cultures of LAB, fermentations with SMC may provide a wider spectrum of functional properties (e.g.,: aroma production; debittering properties; anti-nutritional factors reduction; improving digestibility; health effect). Finally, functional redundancy, plasticity, and robustness is expected from SMC compared to pure cultures which carry a more limited spectrum of functions.

The research approach presented here could be applied to other leguminous-based matrices. Fermentation by SMC of legume-based gels could open new prospects for the design of more sustainable novel foods with more desirable sensorial properties.

## Figures and Tables

**Table 1:** Odorant compounds identified in the pea gels: control sample (not fermented gel) and pea VEGAN gels (after three days of fermentation).

RT	Compounds	CAS Number	Experimental Descriptors (Number of Persons Who Detected the Odorant Compound)	% Frequency of Detection
**Pea control (non-fermented gel)**				
5.85	acetaldehyde	75-07-0	lactic (9) not identified (4)	65
6.52	propanal	123-38-6	vegetal (9) lactic (5)	70
6.8	propan-2-one	64-64-1	roasted/grilled (5), vegetal (4) other (4)	65
7.63	butanal	123-72-8	vegetal (9) lactic (4)	65
8.49	3-methyl butanal	590-86-3	roasted/grilled (10) vegetal (7)	85
10.2	pentanal	110-62-3	roasted/grilled (14)	70
16.88	hexanal	66-25-1	vegetal (20)	100
**Pea-VEGAN**				
5.85	acetaldehyde	75-07-0	lactic (12) not identified (5) other (3),	100
6.8	propan-2-one	64-64-1	roasted/grilled (10) fruity (7)	85
8.28	2-methyl butanal	96.17.3	roasted/grilled (11) vegetal (6)	85
8.49	3-methyl butanal	590-86-3	roasted/grilled (17)	85
**Pea-MEGAN-V**				
8.1	2-methylpropanal	78-84-2	roasted/grilled (11) vegetal (2) lactic (3)	100
11.6	ethyl-2-methylpropanoate	97-62-1	fruity (16)	100
12.1	pentan-2-one	107-87-9	lactic(10) fruity (4) roasted/grilled (1)	94
16.5	ethyl 3-methylbutanoate	108-64-5	fruity (14)	88

**Table 2:** Odorant compounds identified in the mixed gels: control sample (not fermented gel) and mixed MEGAN-A gels (after three days of fermentation).

RT	Compounds	CAS Number	Experimental Descriptors (Number of Persons Who Detected the Odorant Compound)	% Frequency of Detection
**Mixed control (non-fermented gel)**				
6.8	propan-2-one	64-64-1	roasted/grilled (11) fruity (2) vegetal (3)	84
8.28	2-methylbutanal	96.17.3	vegetal (7) not identified(6) lactic (4),	85
8.49	3-methylbutanal	590-86-3	vegetal (8) not identified (5) lactic (3),	85
16.88	hexanal	66-25-1	vegetal (19)	95
**Mixed-MEGAN-A**				
5.85	acetaldehyde	75-07-0	lactic (10) other (3) not identified (3),	80
8.1	2-methylpropanal	78-84-2	roasted/grilled (11) lactic (5)	80
8.28	2-methylbutanal	96.17.3	lactic (8) roasted/grilled (5) fruity (3) not identified (4),	100
8.49	3-methylbutanal	590-86-3	lactic (6) roasted/grilled(5) fruity(2) not identified (4) vegetal (2)	95
9.96	butane-2,3-dione (diacetyl)	431-03-8	butter (20)	100
**Mixed-MEGAN-V**				
8.1	2-methylpropanal	78-84-2	roasted/grilled (12) vegetal (2) lactic(2)	100
8.3	2-methylbutanal or 3-methylbutanal *	96-17-3/590-86-3	roasted/grilled (11) vegetal (1), lactic(1) fruity (1)	93
9.96	butane-2,3-dione	431-03-8	lactic (12) fruity (2),	93
11.6	ethyl-2-methylpropanoate	97-62-1	fruity (14)	93
16.2	methyldisulfanylmethane	624-92-0	fruity (12)	80

* peak co-elution due to analytes abundances.

## Data Availability

Data is contained within the article or Supplementary Materials.

## Supplementary Materials

The following supporting information can be downloaded at: https://www.mdpi.com/article/10.3390/foods11081146/s1, Table S1: Composition of experimental synthetic communities of strains; Table S2: Volatile compounds present in non-fermented and fermented pea and mixed gels. Comparison of quantities (peak area) for three consortia VEGAN, MEGAN-A and MEGAN-V. Within a table line, peak areas with different superscript letters are significantly different (α ≤ 0.05). Green letters refer to pea gels. Blue letters refer to mixed gels.
