# Supplementary material for: Modulation of Metabolome and Overall Perception of Pea Protein-Based Gels Fermented with Various Synthetic Microbial Consortia
Source: Foods. 2022 Apr 15;11(8):1146. doi: 10.3390/foods11081146 (PMC9025280; doi:10.3390/foods11081146)
Supplement: Supplementary file 1 [file foods-11-01146-s001.zip › foods-1631304-supplementary.pdf]

Table S1. Composition of experimental synthetic communities of strains

VEGAN	MEGAN A	MEGAN V	Strains	Origin	Source
	X		*Kluyveromyces lactis,* 3550	Dairy products	UMR Sayfood
	X		*Lactococcus lactis,* S3	Dairy products	UMR Sayfood
X	X		*Lactobacillus rhamnosus,* CNRZ212	Dairy products	UMR Sayfood
X	X		*Diutina catenulata,*Exfcom LD	Dairy products	UMR Sayfood
X	X		*Geotrichum candidum,*ATCC 204307	Dairy products	ATCC
X			*Leuconostoc lactis*, NCW1	Dairy products	UMR Sayfood
X			*Hafnia alvei*, GB001	Dairy products	UMR Sayfood
X			*Brevibacterium antiquum*, CNRZ 918	Dairy products	UMR Sayfood
X			*Brevibacterium casei*, CIP102111	Dairy products	CIP
X			*Yarrowia lipolytica*, CLIB 183	Dairy products	CLIB
		X	*Lactococcus lactis,* NCDO 2118	Legumes	NCDO
		X	*Lactobacillus rhamnosus,* LMG 10772	wine	BCCM/LMG
		X	*Diutina catenulata,* CLIB 1179	Tree	CLIB
		X	*Geotrichum candidum,*CLIB 1154	Cacao	CLIB
		X	*Kluyveromyces lactis*, NCYC 2597	Fermented maize dough	NCYC

TableS2. Volatile compounds present in non-fermented and fermented pea and mixed gels. Comparison of quantities (peak area) for three consortia VEGAN, MEGAN-A and MEGAN-V. Within a table line, peak areas with different superscript letters are significantly different ($\alpha \leq 0.05$). Green letters refer to pea gels. Blue letters refer to mixed gels.

			Control samples (non-fermented)		Fermented samples			
					VEGAN	MEGAN-A	MEGAN-V	
**Volatile compounds**	**Sensory descriptors from literature (Flavornet)***	**CAS number**	**Pea**	**Mixed**	**Pea**	**Mixed**	**Pea**	**Mixed**
**Alcools**								
Ethanol	sweet	64-17-5	nd ^{b}	8,05E+06 ^{c}	nd ^{b}	1,11E+08 ^{a}	1,32E+07 ^{a}	2,55E+07 ^{b}
2-methylpropan-1-ol	wine, solvent, bitter	78-83-1	nd ^{b}	nd	nd ^{b}	nd ^{b}	2,23E+06 ^{a}	3,86E+06 ^{a}
2-methylbutan-1-ol	wine, onion	137-32-6	nd ^{b}	nd	7,09E+06 ^{a}	nd	nd ^{b}	nd
pent-1-en-3-ol	butter, pungent	616-25-1	1,10E+06 ^{a}	8,58E+05 ^{a}	nd ^{b}	nd ^{b}	nd ^{b}	nd ^{b}
propan-1-ol	alcohol, pungent	71-23-8	nd ^{b}	nd	2,28E+06 ^{a}	nd	nd ^{b}	nd
propan-2-ol	alcohol, pungent	67-63-0	5,34E+06 ^{a}	1,10E+07 ^{a}	3,36E+06 ^{b}	nd ^{b}	2,71E+06 ^{b}	1,25E+07 ^{a}
**Aldehydes_Green**								
hexanal	grass, tallow, fat	66-25-1	1,96E+07 ^{a}	1,03E+07 ^{a}	1,54E+06 ^{b}	1,10E+06 ^{c}	3,03E+06 ^{b}	4,85E+06 ^{b}
propanal	solvent, pungent	123-38-6	5,26E+06 ^{a}	4,76E+06 ^{a}	3,69E+05 ^{b}	nd ^{b}	nd ^{b}	nd ^{b}
butanal	pungent, green	123-72-8	2,33E+06 ^{a}	1,11E+06 ^{a}	nd ^{b}	nd ^{b}	nd ^{b}	nd ^{b}
**Aldehydes_Other**								
acetaldehyde	pungent, ether	75-07-0	1,51E+06 ^{a}	nd ^{b}	nd ^{b}	3,62E+0 ^{a}	nd ^{b}	nd ^{b}
2-methylpropanal	pungent, malt	78-84-2	nd ^{b}	nd ^{c}	nd ^{b}	1,69E+07 ^{b}	1,97E+07 ^{a}	2,80E+07 ^{a}
3-methylbutanal	malt	590-86-3	2,89E+06 ^{a}	1,85E+06 ^{b}	2,56E+07 ^{b}	1,26E+07 ^{a}	8,67E+06 ^{a}	1,39E+07 ^{a}
2-methylbutanal	cocoa, almond	96-17-3	1,46E+06 ^{a}	7,11E+05 ^{c}	4,83E+06 ^{b}	2,03E+06 ^{a}	3,52E+05 ^{b}	7,33E+05 ^{b}
(2E,4E)-hexa-2,4-dienal	green, fruity,melon,fresh	142-83-6	nd	nd ^{b}	nd	2,09E+06 ^{a}	nd	nd ^{b}
pentanal	fried	431302-4	9,95E+06 ^{a}	nd	nd ^{b}	nd	nd ^{b}	nd
**Aromatic hydrocarbons**								
2-methyl furan	chocolate	534-22-5	nd	7,29E+05 ^{a}	nd	4,32E+05 ^{b}	nd	1,15E+06 ^{c}
Toluene	paint	108-88-3	1,73E+06 ^{a}	nd	nd ^{b}	nd	nd ^{b}	nd
2-pentylfuran	green bean, butter	3777-69-3	1,79E+06 ^{a}	nd	6,06E+05 ^{b}	nd	nd ^{c}	nd
2-ethylfuran	sweet burnt earthy malty	3208-16-0	4,01E+06 ^{a}	1,79E+06 ^{a}	1,21E+06 ^{b}	nd ^{b}	2,96E+06 ^{a}	2,56E+06 ^{a}
**Sulphur compounds**								
methyldisulfanylmethane	onion, cabbage, putrid	624-92-0	3,54E+06 ^{b}	7,11E+05 ^{a}	5,27E+06 ^{a}	6,13E+05 ^{a}	nd ^{c}	7,85E+05 ^{a}

			Control samples		Fermented samples			
					VEGAN	MEGAN-A	MEGAN-V	
**Volatile compounds**	**Sensory descriptors from literature (Flavornet)***	**CAS number**	**Pea**	**Mixed**	**Pea**	**Mixed**	**Pea**	**Mixed**
**Alkanes**								
pentane	alkane	109-66-0	5,60E+06[b]	2,32E+06[b]	4,58E+06[b]	5,39E+06[a]	1,15E+07[a]	6,20E+06[a]
hexane	alkane	110-54-3	4,76E+06[b]	3,70E+06[a]	6,41E+06[a]	1,24E+06[b]	2,00E+06[c]	3,69E+06[b]
heptane	alkane	142-82-5	1,21E+06[b]	nd[b]	nd[c]	nd[b]	2,81E+06[a]	3,15E+06[a]
Octane	alkane	111-65-9	1,81E+06[a]	2,58E+06[b]	2,80E+06[a]	1,88E+06[b]	nd[b]	5,51E+06[a]
nonane	alkane	111-84-2	nd[b]	nd	2,41E+05[a]	nd	nd[b]	nd
**Alkenes**								
hex-1-ene	not identified	592-76-7	nd	nd[b]	nd	nd[b]	nd	8,92E05[a]
2-methylbuta-1,3-diene	not identified	78-79-5	nd	nd[b]	nd	2,68E+05[a]	nd	nd[b]
**Ketones**								
butan-2-one	ether	78-93-3	7,31E+06[a]	6,99E+06[a]	2,42E+06[b]	1,91E+06[c]	2,79E+06[b]	4,84E+06[b]
propan-2-one	ether	67-64-1	3,40E+07[a]	4,03E+07[a]	1,83E+07[b]	nd[b]	nd[c]	nd[b]
pentan-2-one	ether, fruit	107-87-9	nd[b]	5,96E+06[a]	nd[b]	nd[b]	6,91E+0[a]	nd[b]
heptan-2-one	soap	110-43-0	3,76E+06[a]	1,97E+06[a]	nd[b]	6,11E+05[b]	nd[b]	nd[c]
butane-2,3-dione	butter creamy caramel	431-03-8	nd	nd[c]	nd	1,95E+07[b]	nd	6,99E+07[a]
**Esters**								
ethyl acetate	pineapple	141-78-6	nd[b]	nd[b]	nd[b]	2,88E+06[a]	1,10E+06[a]	1,62E+06[a]
methyl acetate	fruity, banana, sweet	79-20-9	1,90E+06[a]	nd	nd[b]	nd	nd[b]	nd
ethyl-2-methylpropanoate	sour, acid, fruity, berry	97-62-1	nd[b]	nd[b]	nd[b]	nd[b]	1,92E+06[a]	7,33E+05[a]
ethyl 3-methylbutanoate	fruity,pineapple	108-64-5	nd[b]	nd	nd[b]	nd	2,60E+06[a]	nd
**Acid**								
(E)-2-methylpent-2-enoic acid	fruity, acidic,berry	3142-72-1	nd[b]	nd	nd[b]	nd	3,82E+06[a]	nd

* https://www.flavornet.org/flavornet.html
